# Real‐world treatment patterns of adjuvant endocrine therapy and ovarian suppression in premenopausal HR+/HER2+ breast cancer

**DOI:** 10.1002/cam4.7317

**Published:** 2024-06-19

**Authors:** Jasmine S. Sukumar, Sagar Sardesai, Andy Ni, Nicole Williams, Kai Johnson, Dionisia Quiroga, Bhuvana Ramaswamy, Robert Wesolowski, Mathew Cherian, Daniel G. Stover, Margaret Gatti‐Mays, Ashley Pariser, Preeti Sudheendra, Mridula A. George, Maryam Lustberg

**Affiliations:** ^1^ Department of Breast Medical Oncology The University of Texas MD Anderson Cancer Center Houston Texas USA; ^2^ Division of Medical Oncology The Ohio State University Comprehensive Cancer Center Columbus Ohio USA; ^3^ The Ohio State University, College of Public Health Columbus Ohio USA; ^4^ Division of Medical Oncology Rutgers Cancer Institute of New Jersey New Brunswick New Jersey USA; ^5^ Yale Cancer Center New Haven Connecticut USA

**Keywords:** adjuvant therapy, breast cancer, endocrine therapy, HER2‐positive, hormone receptor positive, premenopausal

## Abstract

**Background:**

The optimal adjuvant endocrine therapy (ET) in hormone receptor positive (HR+) and human epidermal growth factor receptor 2 positive (HER2+) premenopausal breast cancer (BC) remains unclear. Moreover, the benefit and clinical indications of ovarian suppression (OS) is poorly elucidated. We described real‐world patterns surrounding choice of ET and clinicopathologic features which predicted treatment with OS in a contemporary cohort of premenopausal women with HR+/HER2+ BC.

**Methods:**

This retrospective analysis included premenopausal patients with nonmetastatic HR+/HER2+ BC from the CancerLinQ Discovery database from January 2010 to May 2020. Women were less than 50 years and received chemotherapy, anti‐HER2 therapy, and ET. They were categorized into 1 of 4 groups based on type of ET prescribed at initiation: aromatase inhibitor (AI) + OS, OS, tamoxifen + OS, or tamoxifen. Multivariable logistic regression assessed associations between clinicopathologic features and OS use.

**Results:**

Out of 360,540 patients with BC, 937 were included. The majority (*n* = 818, 87%) were prescribed tamoxifen, whereas 4 (0.4%), 50 (5.3%), and 65 (6.9%) received OS, tamoxifen + OS and AI + OS, respectively. No clinicopathologic features predicted OS use apart from age; patients <35 years were more likely to receive OS compared with those ≥35 years (odds ratio 2.33, *p* < 0.001).

**Conclusions:**

This is the first real‐world study evaluating ET treatment patterns in HR+/HER2+ premenopausal BC. OS use was uncommon and the majority received tamoxifen as the preferred ET regardless of most clinicopathologic risk factors. Additional research is needed to optimize ET decisions in young women with this distinct BC subtype.

## 1 BACKGROUND

Approximately 10% of all breast cancers are both hormone receptor positive (HR+) and human epidermal growth factor receptor 2 positive (HER2+) and one third to one half of HER2+ breast cancers are also HR+.^1^, ^2^ The concurrent expression with resulting cross‐talk of hormones (estrogen and/or progesterone) and HER2 may distinctly affect cancer outcomes and the natural history of disease in patients with HER2+ breast cancer.^1^, ^3^ Despite treatment advances in the modern era of HER2 targeted therapies for early‐stage breast cancer, there remains a risk for relapse. Therefore, in addition to advancing novel HER2 targeted drugs, understanding how best to target the hormone receptor component is also of great clinical relevance in this subtype.^1^, ^3^ However, the precise additive role of adjuvant endocrine therapy (ET) to HER2 targeted therapy for reduction in recurrence risk as well as the optimal endocrine agent in this setting remains unclear. Data surrounding adjuvant ET in this population is extrapolated from clinical trials in which most analyses focused on the HER2 negative subset of patients. Importantly, patients with HR+/HER2+ disease may have a unique biology compared to those with HR+/HER2‐ breast cancer, and focused investigation surrounding ET best practices is warranted.

While the ideal oral endocrine agent (aromatase inhibitor (AI) or tamoxifen) in HR+/HER2+ breast cancer remains unknown, so does the clinical impact of adding ovarian suppression (OS) in premenopausal women. The combined analysis of data from two large phase III trials investigating adjuvant OS, the Suppression of Ovarian Function Trial (SOFT) and Tamoxifen and Exemestane Trial (TEXT), established a reduction in relapse and improvement in survival when adding 5 years of OS to oral ET (tamoxifen or exemestane) compared to tamoxifen alone in high risk HR+ premenopausal disease.^4^, ^5^, ^6^, ^7^ This benefit was independent of HER2 status; however, the HER2+ cohort was underrepresented. Moreover, these trials were initiated prior to the widespread use of trastuzumab with chemotherapy, which is now standard of care for HER2+ disease. Thus, in the contemporary period of HER2‐targeted agents, the benefit of integration of adjuvant OS into the treatment paradigm for HR+/HER2+ breast cancer has become even less clear.

Oncologists often employ clinical judgment given lack of consensus surrounding ET approaches in this subtype of breast cancer, resulting in differences in treatment patterns. In this study we aimed to describe real‐world treatment patterns pertaining to the choice of adjuvant ET and clinicopathologic features which predicted treatment with OS in premenopausal HR+/HER2+ breast cancer.

## 2 PATIENTS AND METHODS

We performed a multiinstitutional retrospective analysis of premenopausal women with nonmetastatic HR+/HER2+ breast cancer using the American Society of Clinical Oncology (ASCO) CancerLinQ Discovery database, a health technology platform comprising deidentified real‐world data (structured and unstructured) aggregated from Electronic Health Records (EHRs) or supporting data warehouses of over 2.5 million oncology patients across the United States.^8^, ^9^ Electronic health record data was obtained from more than 100 participating academic and community oncology sites.

We included women less than 50 years of age at the time of clinical stage I to III (stage at initial breast cancer diagnosis) invasive breast cancer diagnosis between January 2010 to May 2020. The *DiagnosisActive* table was used to identify primary breast cancer; this table is based on International Classification of Disease Ninth and Tenth Revision (ICD‐9/10) codes. All patients had HR+ (defined as estrogen receptor and/or progesterone receptor positivity by immunohistochemistry [IHC] positive staining >1%) and HER2+ disease (defined as IHC 3+ and/ or fluorescence in situ hybridization [FISH] gene amplification). In addition to the receipt of anti‐HER2 therapy, all eligible patients must have also received chemotherapy (any regimen; possible chemotherapy agents included: doxorubicin, cyclophosphamide, docetaxel, paclitaxel, carboplatin) and ET.

Patients were categorized into one of four groups based on the type of adjuvant ET prescribed at treatment initiation. Options for ET included either: tamoxifen, OS, OS in combination with an AI (anastrozole, letrozole, or exemestane), or OS in combination with tamoxifen. Adjuvant ovarian function suppression was defined as receipt of at least 6 months of medical ovarian function suppression with a gonadotropin‐releasing hormone analog (GnRHa) (either goserelin, leuprolide, or triptorelin) or surgical bilateral oophorectomy. A 6 month criteria was utilized in order to exclude women who may have received medical OS solely for the purpose of ovarian function preservation during receipt of (neo)adjuvant chemotherapy as opposed to for adjuvant ET.

Patients with clinical or pathologic tumor size ≤5 mm (T1a disease) because chemotherapy or OS is typically not recommended for such low risk disease based on current guidelines.^10^ Those with metastatic (stage IV or M1 disease) or stage 0 breast cancer were also excluded. Additionally, any patient who received AI monotherapy (without OS) was excluded, as these women were presumed to be postmenopausal. Demographics, patient and disease characteristics, and treatment history was collected. All antineoplastic treatments (chemotherapy, anti‐HER2 therapy, ET) were identified using generic drug names from the *MedicationOrdered* and *MedicationAdministered* tables.

### 2.1 Statistical analyses

Demographics, clinical characteristics, and treatment modalities were summarized using descriptive statistics and categorized by ET treatment type. Continuous variables were reported descriptively with mean, standard deviation (SD); frequencies and percentages were reported for categorical variables. Multivariable logistic regression was conducted to assess the association between clinicopathologic features (age, race, clinical stage, tumor grade, body mass index (BMI), and noval involvement) and OS use. All data management and analyses were performed by R 4.1.3.^11^

## 3 RESULTS

**FIGURE 1:**
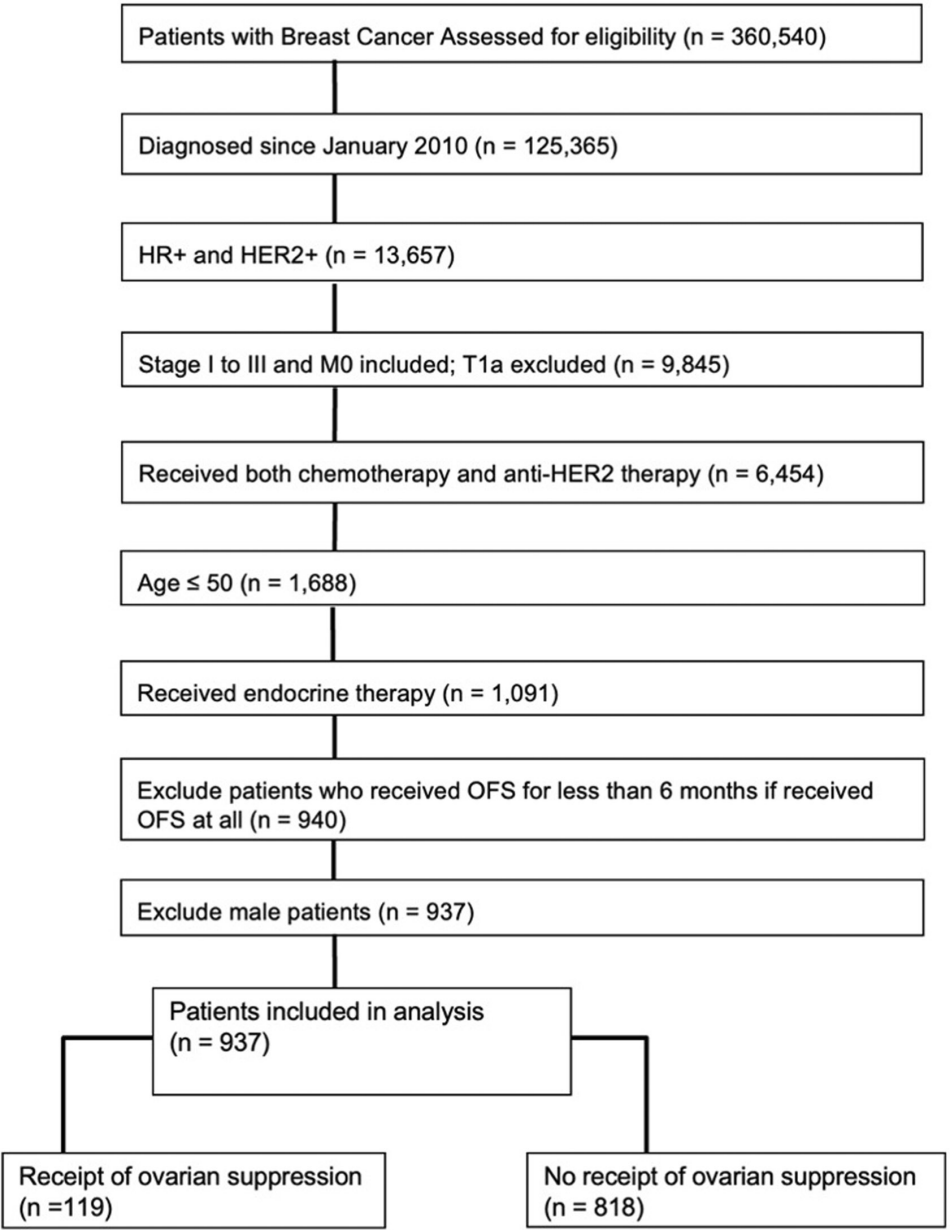
CONSORT diagram for selection of HR+/HER2+ premenopausal breast cancer patients in the CancerLinQ Discovery Database.

Out of 360,540 patients with invasive breast cancer in the database, we identified 937 who met inclusion criteria (Figure 1). Table 1 describes the demographics. treatment, and clinical characteristics of the cohort. The mean age was 41.7 (SD 5.9) years. The majority of patients were white (588, 63%) and had lymph node positive (729, 78%) breast cancer. Among the 937 HER2+ patients, 882 (94%) had IHC positive disease (IHC 3+). HER2 FISH record was missing in the majority, but 55 individuals were recorded to have gene amplification in the database. Nearly all patients (911, 97%) had estrogen receptor positive disease and most (778, 83%) also had progesterone receptor positive disease. All patients received trastuzumab and 497 patients or 53% received trastuzumab in combination with pertuzumab. Most patients (710, 76%) received a non‐anthracycline containing chemotherapy regimen.

**TABLE 1:** Demographic, treatment, and clinical characteristics of study participants by endocrine therapy treatment group.

	Aromatase Inhibitor + ovarian suppression (*n* = 65)	Ovarian suppression (*n* = 4)	Tamoxifen + ovarian suppression (*n* = 50)	Tamoxifen (*n* = 818)	Overall cohort (*n* = 937)
Age (years)					
Mean (SD)	38.4 (5.9)	41.8 (5.5)	38.9 (6.9)	42.2 (5.7)	41.7 (5.9)
Race (*n*, %)					
African American	3 (4.6%)	1 (25%)	6 (12%)	76 (9.3%)	86 (9.2%)
White	41 (63.1%)	0 (0%)	26 (52%)	521 (63.7%)	588 (62.8%)
Other	21 (32.3%)	3 (75%)	18 (36%)	221 (27%)	263 (28.1%)
Body mass index (BMI) (*n*, %)					
<25 kg/m^2^	20 (30.8%)	0 (0%)	12 (24%)	236 (28.9%)	268 (28.6%)
25–30 kg/m^2^	15 (23.1%)	0 (0%)	8 (16%)	129 (15.8%)	152 (16.2%)
≥30 kg/m^2^	5 (7.7%)	3 (75%)	9 (18%)	189 (23.1%)	206 (22.0%)
Unknown	25 (38.5%)	1 (25%)	21 (42%)	264 (32.3%)	311 (33.2%)
ER positive (*n*, %)	65 (100%)	3 (75%)	50 (100%)	793 (96.9%)	911 (97.2%)
PR positive (*n*, %)	55 (84.6%)	3 (75%)	44 (88%)	676 (82.6%)	778 (83%)
Clinical stage (*n*, %)					
1	30 (46.2%)	0 (0%)	19 (38%)	314 (38.4%)	363 (38.7%)
2	28 (43.1%)	3 (75.0%)	17 (34%)	368 (45%)	416 (44.4%)
3	7 (10.8%)	1 (25%)	14 (28%)	136 (16.6%)	158 (16.9%)
Tumor grade (*n*, %)					
1	1 (1.5%)	0 (0%)	0 (0%)	18 (2.2%)	19 (2.0%)
2	11 (16.9%)	1 (25%)	12 (24.0%)	172 (21.0%)	196 (20.9%)
3	24 (36.9%)	2 (50%)	18 (36%)	224 (27.4%)	268 (28.6%)
Unknown	29 (44.6%)	1 (25%)	20 (40%)	404 (49.4%)	454 (48.5%)
Nodal involvement (*n*, %)					
N0	11 (16.9%)	0 (0%)	10 (20%)	187 (22.9%)	208 (22.2%)
≥N1	54 (83.1%)	4 (100%)	40 (80%)	631 (77.1%)	729 (77.8%)
Anti‐HER2 therapy (*n*, %)					
Trastuzumab	65 (100%)	4 (100%)	50 (100%)	818 (100%)	937 (100%)
Pertuzumab	53 (81.5%)	4 (100%)	33 (66%)	407 (49.8%)	497 (53%)
Anthracycline use (*n*, %)	8 (12.3%)	0 (0%)	13 (26.0%)	206 (25.2%)	227 (24.2%)

**FIGURE 2:**
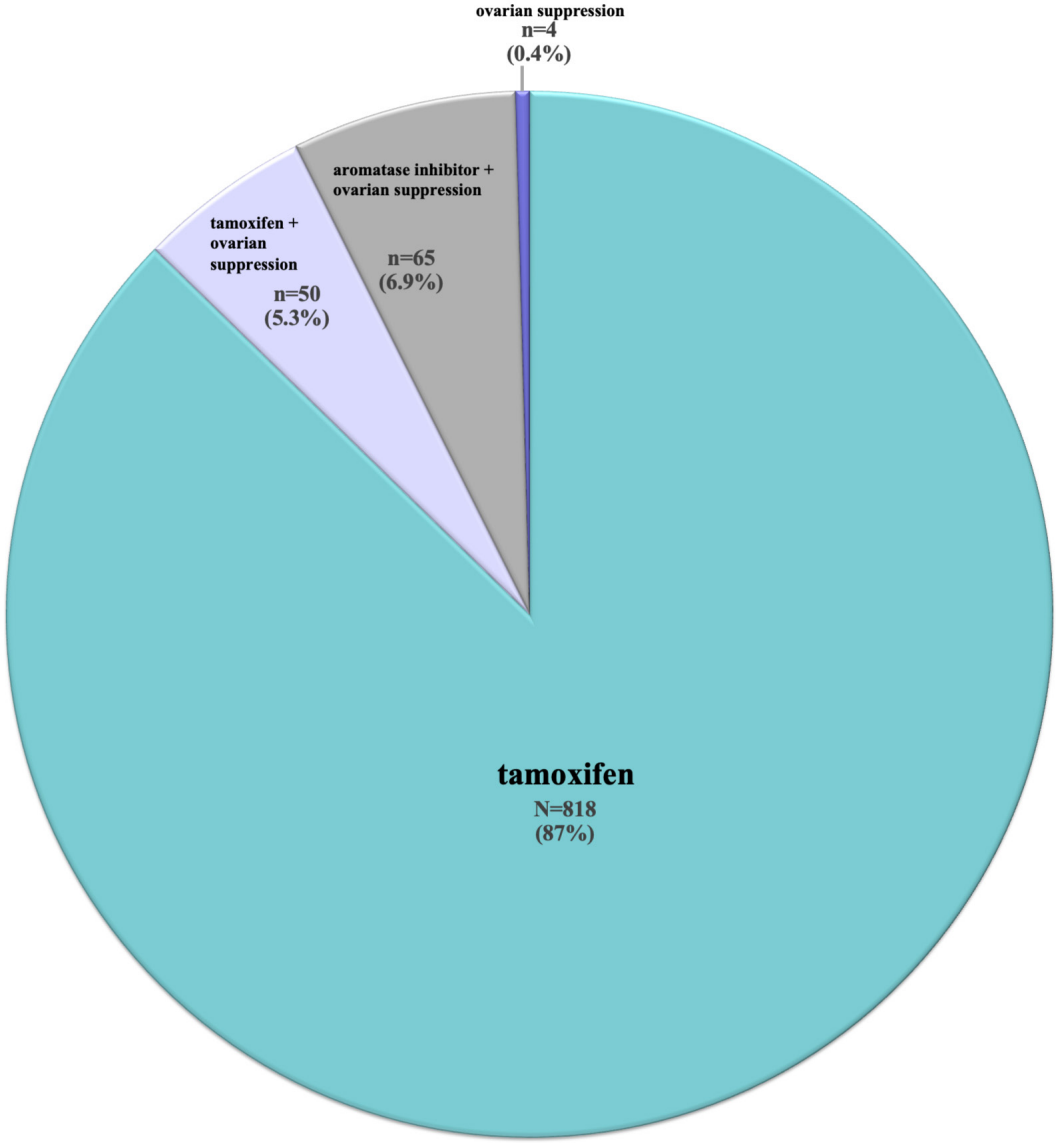
Premenopausal Women with HR+/HER2+ Breast Cancer (n = 937) in the CancerLinQ cohort categorized by endocrine therapy treatment group.

Most patients (*n* = 818, 87%) were prescribed tamoxifen as the initial adjuvant ET whereas only 4 (0.4%), 50 (5.3%), and 65 (6.9%) received OS, tamoxifen with OS, or an AI with OS, respectively (Figure 2). Among the 119 patients who received OS, 117 (98%) received medical OS whereas only 2 (1.7%) received surgical OS. Moreover, the majority (112, 94.1%) of the 119 patients who received OS started it after year 2015. No clinicopathologic features predicted OS use apart from age; patients <35 years were more likely to receive OS compared with those ≥35 years (Odds Ratio 2.33, 95% CI 1.47, 3.70, *p* < 0.001) (Table 2). BMI (obese vs. nonobese), race (white vs. African American), stage (3 vs. 1 or 2), tumor grade (3 vs. 1 or 2), and nodal involvement did not predict the use of OS (Table 2).

**TABLE 2:** Multivariable logistic regression model of clinicopathologic characteristics to predict use of Ovarian Suppression.

Clinicopathologic variable	Odds ratio (95% CI)	*p* value
Age: <35 years vs. ≥35	2.33 (1.47, 3.70)	***p* < 0.001**
Race: white vs. African American	0.92 (0.45, 1.90)	*p* = 0.829
Clinical stage: 3 vs. 1 and 2	1.13 (0.67, 1.90)	*p* = 0.653
Tumor grade: 3 vs. 1 and 2	1.36 (0.79, 2.33)	*p* = 0.263
Nodal involvement: ≥N1 vs. N0	1.27 (0.76, 2.14)	*p* = 0.366
Body mass index (BMI) ≥ 30 vs. <25 kg/m^2^	0.72 (0.38, 1.35)	*p* = 0.308

The bold value represents the variable which showed statistical significance.

## 4 DISCUSSION

To our knowledge, this is the first study evaluating the use of adjuvant ET in a real‐world population of premenopausal women with HR+/HER2+ breast cancer. Moreover, our study uniquely describes practice patterns with OS treatment. We found that the use of OS as part of adjuvant ET was quite uncommon; it was the initial endocrine regimen of choice in only 13% of patients. Additional exploration in other cohorts and databases as well as prospective research is critical to further elucidate OS decision making and practice patterns in premenopausal HR+/HER2+ breast cancer.

The addition of OS to oral ET was surprisingly not associated with several. High risk clinicopathologic features as one would expect. For example, node positivity and stage III disease were not associated with the use of OS despite guidelines which recommend its administration in patients with higher‐risk HR+ disease.^12^ However, age < 35 years was one factor which did predict its use, and this practice is consistent with findings from the SOFT and TEXT studies, where younger age at diagnosis was associated with greater breast cancer risk as well as clinical benefit from OS.^5^ This is also consistent with a recent analysis from the Early Breast Cancer Trialists' Collaborative Group (EBCTCG), where recurrence reductions were significantly larger among premenopausal women <45 years (event rate ratio 0.63) compared with women 45–54 years (event rate ratio 0.84).^13^ Our findings suggest the wide variability in real‐world practice patterns surrounding OS. Namely, the heterogenous settings for utilizing it without clear clinical predictors suggest that the optimal setting for OS in this particular subtype of breast cancer is not well understood. Moreover, the rates of each oral endocrine agent to combine with OS were relatively similar (tamoxifen *n* = 50 or 42.0%; AI, *n* = 65 or 54.6%), highlighting this choice remains heterogenous in practice.

Previous studies have reported on HR+/HER2+ breast cancer and relative efficacy by adjuvant ET type, including the incorporation of OS, albeit with conflicting results.^1^, ^14^, ^15^, ^16^ Moreover, these were either exploratory analyses or included a small subset with limited events and few conclusions can be drawn to inform best practices. One of the largest includes the results of the combined SOFT and TEXT population, which showed that women with premenopausal HR+ disease, regardless of HER2 status, had improved disease free survival (DFS) when treated with 5 years of OS given in addition to oral ET (AI or tamoxifen) compared to tamoxifen alone.^4^, ^5^, ^6^, ^7^ While women with HR+/HER2+ breast cancer were underrepresented (*n* = 695, 12%), a subgroup analysis in this cohort demonstrated an improved DFS with the addition of OS to an AI (8 year DFS 75.2% exemestane plus OS vs. 68.3% tamoxifen, HR 0.75, 95% CI 0.45–1.22) or tamoxifen (8 year DFS 85.4% tamoxifen plus OS vs. 68.3% tamoxifen, HR 0.41, 95% CI 0.22–0.75).^4^ In a recently published long‐term follow up of SOFT and TEXT, cancer outcomes were numerically in favor of tamoxifen over an AI as the oral ET partner in combination with OS in HER2+ disease (13 year DRFI 87.9% tamoxifen + OS vs. 80.4% AI + OS; absolute difference−7.5 (95% CI‐13.8‐1.2)).^7^

Several studies beyond SOFT and TEXT have also expored ET type in relation to cancer outcome in HR+ HER2‐ disease. Another example is the recent analysis of HR+/HER2+ breast cancer patients from the phase III ShortHER study (*n* = 784), of which 40% (*n* = 309) were premenopausal.^14^ In contrast to the SOFT/TEXT population, treatment with an AI was associated with improved 8‐year DFS compared with other ET choices (HR 1.64, 95% CI 1.07–2.52, *p* = 0.025).^14^ However, like SOFT/TEXT, the use of medical OS with a GnRHa was associated with improved DFS (85.2 vs. 62.6%, HR 0.41, p = 0.019, 95% CI 0.19–0.88).^14^ Another meta‐analysis of six large trials (TEAM, ATAC, BIG 1–98, SOFT, TEXT, ALTTO) in 5390 women (45% premenopausal) with HR+/HER2+ breast cancer conversely showed no significant DFS difference between an AI and tamoxifen. However, conclusions regarding OS were not possible because only patients in SOFT/TEXT received this, whereas those in the other 4 studies did not.^1^ In another population‐based study of Dutch patients with HR+/HER2+ disease from the Netherlands Cancer Registry, there was again no significant difference in outcomes between an AI over tamoxifen in the subgroup of 326 premenopausal women. Moreover, a numerical but non‐significant survival benefit was observed with the use of OS (HR 0.8, 95% CI 0.49–1.31, p = 0.38).^16^

Our study benefited from engaging the ASCO CancerLinQ database, which provided a sizeable cohort of patients with characteristics of a real world oncology population. The inclusion of several cancer centers, both community and academic, ensured a representative population of cancer patients nationwide. Moreover, our findings are unique in that it encompassed modern treatment regimens for HER2+ disease in line with current clinical guidelines.^10^ Namely, all patients received chemotherapy and anti‐HER2 directed therapy. Additionally, most individuals did not receive an anthracycline. This is consistent with the contemporary and generally preferred treatment approach for HER2+ disease, as an anthracycline containing chemotherapy regimen added to anti‐HER2 therapy does not improve treatment efficacy but does increase cardiotoxicity and risk of treatment associated acute myeloid leukemia compared with an anthracycline free regimen.^10^, ^17^ In contrast, most other published data surrounding ET in HR+/HER2+ disease included patients prior to the wide use of trastuzumab/pertuzumab or included more individuals who received a non‐standard anthracycline containing chemotherapy backbone.^1^, ^5^, ^14^, ^16^ For example, only 54% of patients with HER2+ disease in the combined SOFT and TEXT population received anti‐HER2 directed therapy, which is now recommended for nearly all patients with HER2+ breast cancer.^5^, ^10^

Our study was also unique in that we concentrated on the patients who were presumably premenopausal at the time of adjuvant ET decision‐making. In total, we excluded 227 patients less than 50 years who were prescribed adjuvant AI monotherapy, as these women would be considered post‐menopausal (either because of baseline post‐menopausal status or chemotherapy‐induced menopause). We intended to focus on the patients who remained premenopausal after receipt of chemotherapy given that the optimal approach to ET in this specific group of patients with HER2+ disease remains a challenge in clinical practice. However, we acknowledge that the OS uptake rates could have been affected by the presence or absence of chemotherapy‐induced menopause, which could not be ascertained within the constraints of this database. This is relevant given that breast cancer recurrence reductions from OS are suggested to be larger among women established to be premenopausal prior to OS than among those whose menopausal status is uncertain after chemotherapy. This is supported by data from a large meta‐analysis by EBCTCG comprising 14,993 women across 25 trials (event rate ratio = 0.70 for confirmed premenopausal vs. 0.91 for unknown menopausal status after chemotherapy, *p* = 0.0003).^13^

This study is not without limitations. As with all large real‐world datasets, missing data are a drawback. Menopausal status could not directly be obtained and as a proxy we defined premenopausal status with an age cutoff of less than 50 years. Additionally, the database did not include information on adverse events. However, it is well established that compared with tamoxifen alone, OS is associated with a considerable increase in toxicities such as sexual dysfunction, menopause symptoms, and decreased quality of life, and this may impact therapeutic decisions.^12^ Namely, patient tolerability and shared decision making are key factors which can influence choice of ET on an individualized basis. However, collecting these type of data was not feasible due to the limitations of any retrospective study involving a large national database. We were also not able to analyze ET categories in relation to cancer outcomes. In this case, capturing certain details of adjuvant ET such as treatment duration or accounting for switches in ET type was not feasible with the dataset. Without this information no reliable conclusions can be drawn regarding cancer outcomes. However, as summarized, several prior studies in both clinical trial and real‐world cohorts have reported on the comparative efficacy by ET type in HR+/HER2+ disease. Moreover, the inability to collect certain data (eg. switches in ET type, development of chemotherapy‐induced menopause) based on the retrospective design could have led to a potential bias with regards to the estimation OS usage. Lastly, this study drew upon a population starting at year 2010 to improve the sample size and investigate temporal changes in OS usage. However, the initial results of SOFT were not published until 2015, and this likely impacted the observed ET practice patterns.^4^ Namely, the benefit of OS was less clear prior to 2015; this could explain its uncommon receipt between year 2010–2015 but increased use following this time period.

## 5 CONCLUSION

In a large real‐world cohort of premenopausal HR+/HER2+ breast cancer patients in the United States, the use of OS as part of adjuvant ET was uncommon and most women were prescribed tamoxifen alone as the preferred ET regimen regardless of several high risk clinicopathologic features. Further investigation is warranted to characterize the potential added benefit of OS for risk reduction in the context of modern‐day treatments for premenopausal HR+/HER2+ breast cancer. This will better inform a personalized approach for optimal outcomes. This is also an area of high clinical relevance as we continue to advance our knowledge of de‐escalated chemotherapy‐sparing and more targeted regimens for certain cases of HER2+ disease (NCT04569747, NCT03161353). Thus, optimizing the approach to anti‐estrogen therapy to further tailor treatment decisions in this breast cancer subtype is a priority.

## AUTHOR CONTRIBUTIONS

**Jasmine S. Sukumar:** Conceptualization (lead); formal analysis (equal); investigation (lead); methodology (lead); project administration (lead); writing – original draft (lead); writing – review and editing (equal). **Sagar Sardesai:** Conceptualization (equal); formal analysis (equal); investigation (equal); methodology (equal); project administration (equal); writing – review and editing (equal). **Andy Ni:** Data curation (lead); formal analysis (lead); investigation (equal); project administration (equal); writing – review and editing (lead). **Nicole Williams:** Writing – review and editing (supporting). **Kai Johnson:** Writing – review and editing (equal). **Dionisia Quiroga:** Writing – review and editing (supporting). **Bhuvana Ramaswamy:** Writing – review and editing (equal). **Robert Wesolowski:** Writing – review and editing (equal). **Mathew Cherian:** Writing – review and editing (equal). **Daniel G. Stover:** Writing – review and editing (equal). **Margaret Gatti‐Mays:** Writing – review and editing (equal). **Ashley Pariser:** Writing – review and editing (equal). **Preeti Sudheendra:** Writing – review and editing (equal). **Mridula A. George:** Conceptualization (equal); formal analysis (equal); investigation (equal); methodology (equal); writing – review and editing (equal). **Maryam Lustberg:** Conceptualization (lead); formal analysis (supporting); investigation (lead); methodology (lead); project administration (equal); writing – review and editing (lead).

## FUNDING INFORMATION

The authors declare that no funds, grants, or other support were received during the preparation of this manuscript.

## CONFLICT OF INTEREST STATEMENT

Jasmine Sukumar, Andy Ni, Kai Johnson, Dionisia Quiroga, Bhuvana Ramaswamy, Robert Wesolowski, Daniel Stover, Mathew Cherian, Margaret Gatti‐Mays, Ashley Pariser, Preeti Sudheendra, and Nicole Williams have no competing interests to declare. Mridula George reports research funding from Incyte and Oncolytics. Maryam Lustberg reports consulting fees from AstraZeneca, Lily, Pfizer, Gilead, and Novartis. Sagar Sardesai reports consulting fees and/or research funding from Gilead, Stemline, and AstraZeneca.

## ETHICS STATEMENT

This is a retrospective study using the CancerLinQ Discovery Database. Thus, no ethical approval or patient consent is required.

## Data Availability

The datasets generated during and/or analyzed during the current study were obtained from the CancerLinQ Discovery Database. The authors confirm that data supporting the findings of this study are available within the article and/or the tables.
